# Comparison of the NIST and PTB Air-Kerma Standards for Low-Energy X-Rays

**DOI:** 10.6028/jres.114.023

**Published:** 2009-12-01

**Authors:** Michelle O’Brien, Ludwig Bueermann

**Affiliations:** National Institute of Standards and Technology, Gaithersburg, MD 20899; Physikalisch-Technische Bundesanstalt, Braunschweig, Germany

**Keywords:** air kerma, free-air ionization chamber, mammography qualities, primary standard, reference radiation qualities, x-ray calibration, x-rays

## Abstract

A comparison has been made of the air-kerma standards for low-energy x rays at the National Institute of Standards and Technology (NIST) and the Physikalisch-Technische Bundesanstalt (PTB). The comparison involved a series of measurements at the PTB and the NIST using the air-kerma standards and two NIST reference-class transfer ionization chamber standards. Results are presented for the reference radiation beam qualities in the range from 25 kV to 50 kV for low energy x rays, including the techniques used for mammography dose traceability. The tungsten generated reference radiation qualities, between 25 kV and 50 kV used for this comparison, are new to NIST; therefore this comparison will serve as the preliminary comparison for NIST and a verification of the primary standard correction factors. The mammography comparison will repeat two previously unpublished comparisons between PTB and NIST. The results show the standards to be in reasonable agreement within the standard uncertainty of the comparison of about 0.4 %.

## 1. Introduction

An indirect comparison was made between the air-kerma primary standards for low-energy x rays of the National Institute of Standards and Technology (NIST) and the Physikalisch-Technische Bundesanstalt (PTB). The measurements were conducted at both laboratories between January 2006 and September 2008 using the tungsten reference radiation qualities in the range from 10 kV to 50 kV recommended by the Consultative Committee for Ionizing Radiation (CCRI) [1] and various mammography qualities. Two NIST reference-class ionization chambers were used as transfer instruments for the comparison, one of which has been used for measurement comparisons between NIST and PTB for mammography twice between 1995 and the present.

## 2. Determination of the Air-Kerma Rate

For a free-air ionization chamber standard with measuring volume *V*, the air-kerma rate, *K*, is determined by the equation

$$\dot{K}=\frac{I}{\rho_{\text{air}}V}\frac{W_{\text{air}}}{e}\frac{1}{1-g_{\text{air}}}\prod_{i}K_{i},$$ (1)

where

*I* / *ρ*_air_*V*: is the mass ionization current measured by the standard,
*W*_air_: is the mean energy expended by an electron of charge *e* to produce an ion pair in dry air,
*g*_air_: is the fraction of the initial electron energy lost by bremsstrahlung production in air, and
∏ *k*_i_: is the product of the correction factors to be applied to the standard.

The values for the physical constants used in the determination of the air-kerma rate are given in Table 1.

## 3. Characteristics of Chambers

### 3.1 Description of Air-Kerma Standards

Two of the air-kerma standard chambers used in the comparison are parallel-plate free-air ionization chambers and the third standard is a variable volume cylindrical chamber. The measuring volume *V* is defined by the diameter of the chamber aperture and the length of the collecting plate. The PTB air-kerma standard is described in Ref. [2]. The NIST Ritz and Attix chambers are described in Refs. [3–6]. The main dimensions, the measuring volume and the polarizing voltage for each chamber are given in Table 2a.

### 3.2 Description of Transfer Ionization Chambers

Two NIST transfer chambers were used for the comparison. The chambers used for the low-energy x rays were the Exradin A15 SN 103 and the Exradin A11 SN 114. Each was calibrated at the NIST before and after a series of calibrations at the PTB between January 2006 and September 2008. The transfer standards are constructed with a wall of air-equivalent plastic. Table 2b contains the physical characteristics of the transfer chambers. A collecting voltage of 300 V was applied to each standard. Measurements were made using both polarities at each laboratory.

## 4. Comparison Details

### 4.1 Irradiation Facilities and Reference Radiation Qualities

The radiation qualities in the energy range between 10 kV and 50 kV are those recommended by CCRI [1] and are given in Table 3a for both NIST and PTB. NIST developed the NIST equivalent CCRI reference radiation qualities to allow for direct comparisons with the Bureau International des Poids et Mesures (BIPM). In past comparisons, the NIST had to interpolate values for radiation qualities with the same energy but different filtration and half-value layers (HVL). The parameters of the mammography reference radiation qualities are listed in Table 3b. The four reference radiation qualities which are not supported by the standards used in this comparison both at the PTB and the NIST calibration facilities are the NIST qualities at 23 kV and 40 kV and the PTB qualities at 10 kV and 20 kV. The associated data for these techniques are included in this report for documentation completeness; however no comparison results are presented. Initially the 10 kV reference technique was to be included in the comparison results, but in the course of this comparison it was determined that the Ritz chamber is unsuitable for the soft 10 kV reference radiation quality. A different NIST primary standard, the Lamperti chamber [7] has been previously compared [8] at the BIPM providing traceability for 10 kV. Due to a new measurement capability at NIST, the Lamperti chamber will be primarily used in a different facility. The Lamperti standard was not included in this comparison.

The NIST x-ray measurements were made using primary standard free-air ionization chambers, the Ritz in the NIST 100 kV tungsten calibration facility range and the Attic in the mammography facility. For the NIST 100 kV range, the x-ray source is a 100 kV x-ray generator with a metal-ceramic x-ray tube, supplied by Pantak and Comet^1^. In the NIST mammography facility the x-ray source is a Gulmay generator and a Lohmen Mo anode tube. Both x-ray generators are high-frequency, highly stabilized voltage sources. Both tubes have a 1 mm beryllium window. The materials used for the filtration and for the measurement of HVL were at least 99.99 % pure with thicknesses known with an uncertainty of 0.01 mm. The high voltage was verified through the use of an invasive voltage divider custom made by Pantak.

The PTB x-ray air kerma measurements were made using the PK100 primary standard free-air ionization chamber. The PTB mammographic reference radiation qualities are produced with a unipolar x-ray tube of type Philips PW2185/Mo with a Mo anode angle of 26° combined with a constant potential generator. The in-herent filtration is 1 mm beryllium and the molybdenum filtration is 0.03 mm. Radiation qualities are established for tube voltages in the range from 20 kV to 50 kV. The PTB CCRI radiation qualities are also realized with a unipolar x-ray tube of type Comet MXR 160/0.4–3.0 with a W anode angle of 20° combined with a constant potential generator. The inherent filtration is 1 mm beryllium. For both x-ray facilities the high voltage was measured invasively with a voltage divider manufactured and calibrated at PTB. A transmission-type monitor chamber manufactured at TB was used at both facilities to normalize the x-ray output.

### 4.2 Correction Factors

Although free-air chambers are designed to minimize or eliminate corrections to the measured ionization current, certain corrections are unavoidable. The correction factors applied to each free-air chamber and the associated uncertainties are listed in Tables 4 to 6c. A correction must be made for the attenuation of the x-ray fluence along the air path between the reference plane and the center of the collecting volume. The correction factor *k*_a_ is calculated using the measured air-attenuation coefficients *μ*_air_, according to

$$K_{a}=\exp(\mu_{\text{air}}L).$$ (2)

The effective attenuation path *L* varies with the temperature and pressure of the air in the chamber and so the values for *k*_a_ are corrected for this effect. All ionization measurements are also corrected for the temperature and pressure of the ambient air between the radiation source and the reference plane.

All measured ionization currents using the free-air chamber standards are corrected for ion recombination, *k*_s_. The ionization currents measured with the transfer standards are not corrected for ion recombination. However, since the air-kerma rates used at both facilities are low, minimal volume recombination occurs in the transfer chambers. The standard chambers are corrected for the humidity effect *k*_h_, which is taken as 0.998 for NIST and PTB standards for all reference radiation qualities.

### 4.3 Chamber Positioning and Measurement Procedure

Each calibration was made by alternating between the transfer chambers and the standard free-air chamber for the x-ray measurements. At both laboratories, alignment on the beam axis was measured to an accuracy of around 0.1 mm, and this position was reproducible to better than 0.01 mm as observed by an alignment telescope. No correction was applied for the radial non-uniformity of any beam. The reference plane for each x-ray standard chamber was positioned at 500 mm for the tungsten work and 1000 mm for the mammography work. At NIST, the beam diameter in the reference plane was 70 mm for the beams measured with the Ritz chamber and 80 mm for those measured with the Attix chamber. The beam diameter in the reference plane for the PTB x-ray measurements was 83 mm. The background current was measured before and after each series of ionization current measurements and a correction made based on the mean of these leakage measurements. The background current-to-signal ratios varied for the low rate attenuated mammography beams and the higher rate tungsten and unfiltered mammography beams. The background current-to-signal ratios were between 1 % and less than 0.01 %. For all x-ray chambers at the NIST, a total of not less than 10 measurement sets with an integration time of 120 s, 90 s or 60 s were made, except for the data for the mammography positive charge sets. Due to equipment failure, only two sets of three 120 s measurements were made for the mammography positive charge data. The standard deviations (Type A uncertainty components) of the calibration coefficient measurements for the CCRI (NIST-BIPM) beams were typically 0.1 % for the NIST transfer chambers. The standard deviations of the calibration coefficient measurements for the NIST mammography beams were typically less than 0.5 % for the NIST transfer chambers. The measurement procedure at PTB is similar to those at NIST. The leakage charge was measured before and after a series of five repeated charge measurements with the x-ray beam turned on and the mean of both leakage charge measurements was subtracted. The integration times were 60 s and 100 s depending on the actual chamber currents. A transmission-type monitor chamber manufactured at PTB was used to normalize the x-ray output. The standard deviations of the calibration coefficients of both transfer chambers were always less than 0.1 %. The leakage-to-signal ratio of the A11 and A15 chamber was less than 0.5 % and 0.2 % in the attenuated mammography beams, respectively, and less than 0.1 % in every other case.

All transfer ionization chamber current measurements were normalized to 293.15 K and 101 325 Pa. The transfer chamber currents were not corrected for humidity. The humidity is monitored and recorded at both facilities. The PTB laboratory humidity is not controlled because it varies by no more than between 30 % and 60 % and this is taken into account by an additional uncertainty in the humidity correction factor. The NIST laboratory average humidity is 30 %.

## 5. Measurement Uncertainties

The uncertainties associated with the primary standards are given in Table 7. The NIST uncertainties were evaluated according to Ref. [9]. The uncertainties associated with the calibration of the transfer ionization chambers at the NIST and at the PTB are listed in Table 8. The uncertainty of the comparison results is given in Table 9. The comparison uncertainty calculation removed the correlations due to Type B uncertainties from the physical constants and the correction for humidity. Correlations also exist in the values for *k*_e_, *k*_sc_ and *k*_fl_, since both laboratories use values derived from the same Monte Carlo calculations [10]. These are accounted for by taking half the uncertainty value for each component at each laboratory, in accordance with the analysis made for the degrees of equivalence appearing in Ref. 11. All other correction factors are assumed to be uncorrelated.

## 6. Results and Discussion

The results for the transfer ionization chamber calibrations at both laboratories are shown in Tables 10a and 10b. The average of the calibration coefficients collected at both polarities was determined for each chamber and then used to determine the comparison ratios. For each transfer chamber and each radiation quality, the ratio of the calibration coefficients *N_K_*_, NIST_ / *N_K_*_, PTB_ is evaluated, as shown in Table 11, as well as the mean of the ratios. Comparison results are not included for the 10 kV technique, because it was verified through the course of the comparison that the Ritz chamber, as currently used at NIST with the 7 cm collecting plate, is not well suited for the soft 10 kV reference radiation qualities. Although correction factors have previously been determined for the 10 kV beam using the Ritz chamber, more work would be required to determine the appropriateness of the replacement of the Lamperti chamber with the Ritz chamber for measurement traceability at 10 kV. The agreement of the Ritz to the PTB PK100 standard for 10 kV is on the order of 2.5 %, which is the same agreement previously determined between the two NIST low energy standards, the Lamperti and the Ritz chamber. This disagreement has never been a concern because until recently the Lamperti chamber was readily available to provide traceability for 10 kV. The 10 kV reference techniques at NIST are generally only used for comparison measurement work at NIST and rarely requested for routine calibrations. Unless there is increased demand or interest in the 10 kV technique, no further investigation into the limitations of the Ritz chamber will transpire.

Agreement of 0.5 % can be achieved for the reference radiation qualities used for this comparison. Agreement of 0.2 % was achieved for the 0.03 mm Mo filtered mammography techniques, while the mammography reference radiation qualities filtered with the 2 mm of aluminum compared on the 0.5% level. The comparison results for mammography techniques demonstrated consistency between NIST and PTB from the previous two series of unpublished measurements performed since 1995. The comparison results for the 25 kV, 30 kV and 50 kV tungsten reference radiation qualities serve as verification to the changes and additions to the reference radiation qualities maintained at the NIST. The correction factors for the Ritz chamber will be verified and perhaps reevaluated through some calculations supported by recently acquired spectral measurements.

## Figures and Tables

**Table 1:** Physical constants used in the determination of the air-kerma rate

Physical constant	Value	Relative standard uncertainty (%)
ρ_air_^a^	1.293 kg m^−3^	0.01
*W*_air_/*e*	33.97 J C^−1^	0.15
1 – *g*_air_	1.0000	0.01

a Density of dry air at 273.15 K and 101 325 Pa.

**Table 2a:** Main characteristics of the primary standards used in the comparison

Characteristic	NIST Ritz	NIST Attix	PTB PK100
Air-path length / cm	12.74	21.27	9.72
Plate separation / cm	9.0	Variable	23.4
Collecting plate length / cm	7.003	Variable	2.0021
Aperture diameter / cm	1.0	1.0 or 0.5	2.0008
Measuring volume / cm^3^	5.502	Variable	6.2947
Polarizing voltage / V	−5000	−2500	6000

**Table 2b:** Main characteristics of ionization chambers used as transfer standards

Model	Serial number	Type	Sensitive volume	Collector diameter
A11	114	PLANAR	0.62 cm^3^	20 mm
A15	103	PLANAR	2.46 cm^3^	20 mm

**Table 3a:** Characteristics of the reference radiation qualities used for the comparison at a measurement position of 50 cm

Reference radiation	Gernerating potential	Additional filtration		Half-value layer (HVL)	Air-kerma rate
	kV	mm A1	mm Cu	mm A1	mGy s^−1^
PTB					

BIPM10	10	0		0.033	0.28
BIPM25	25	0.374		0.239	0.28
BIPM30	30	0.208		0.163	0.42
BIPM50a	50	4.00		2.291	0.19
BIPM50b	50	1.00		1.065	0.28

NIST					

BIPM10	10	0		0.037	2.60
BIPM25	25	0.373		0.240	1.40
BIPM30	30	0.203		0.167	4.20
BIPM40	40	3.97	0.208	2.649	0.043
BIPM50a	50	3.97		2.291	0.730
BIPM50b	50	1.07		1.038	3.20

**Table 3b:** Characteristics of the mammography reference radiation qualities used for the comparison at a measurement position of 100 cm

Reference radiation	Tube voltage	Additional filtration	Half-value layer (HVL)	Air-kerma rate
	(kV)	(mm)	(mm A1)	mGy s^−1^
PTB				

MMV 20	20	0.03 Mo	0.22	0.555
MMV 25	25	0.03 Mo	0.29	0.108
MMV 28	28	0.03 Mo	0.32	1.070
MMV 30	30	0.03 Mo	0.33	1.030
MMV 35	35	0.03 Mo	0.37	1.070
MMH 20	20	0.030 Mo + 2.0 Al	0.46	0.013
MMH 25	25	0.030 Mo + 2.0 Al	0.56	0.062
MMH 28	28	0.030 Mo + 2.0 Al	0.61	0.069
MMH 30	30	0.030 Mo + 2.0 Al	0.64	0.071
MMH 35	35	0.030 Mo + 2.0 Al	0.73	0.070

NIST				

Mo/Mo23	23	0.032 Mo	0.271	0.330
Mo/Mo25	25	0.032 Mo	0.296	0.460
Mo/Mo28	28	0.032 Mo	0.332	0.680
Mo/Mo30	30	0.032 Mo	0.351	0.400
Mo/Mo35	35	0.032 Mo	0.392	0.840
Mo/Mo25x	25	0.030 Mo + 2.0 Al	0.566	0.018
Mo/Mo28x	28	0.030 Mo + 2.0 Al	0.626	0.038
Mo/Mo30x	30	0.030 Mo + 2.0 Al	0.660	0.055
Mo/Mo35x	35	0.030 Mo + 2.0 Al	0.748	0.120

**Table 4:** Correction factors used in the comparison for the NIST Ritz standard

Correction factor	Generating potential (kV)					Relative standard uncertainty	
	25	30	40	50a	50b	Type A	Type B
Air attenuation *k*_a_^a^	1.039	1.054	1.005	1.006	1.012	0.01	0.01
Scattered radiation *k*_sc_	0.9966	0.9967	0.9976	0.9974	0.9952		0.07
Electron loss *k*_e_	1.000	1.000	1.000	1.005	1.001		0.05
Ion recombination *k*_s_	1.000	1.000	1.000	1.000	1.000	0.03	
Fluorescence *k*_fl_	0.9961	0.9962	0.99828	0.9981	0.9976		0.03
Aperture transmission *k*_l_	1.000	1.000	1.000	1.000	1.000		0.04
Field distortion *k*_d_	1.000	1.000	1.000	1.000	1.000		0.1
Wall transmission *k*_p_	1.000	1.000	1.000	1.000	1.000		0.01
Humidity *k*_h_	0.998	0.998	0.998	0.998	0.998		0.03

a These are nominal values for *T* = 293.15 K and *p* = 101 325 Pa. Each measurement is corrected using the air temperature and pressure at time of measurement.

**Table 5a:** Correction factors used in the comparison for the NIST Attix mammography standard

Correction factor	Reference radiation qualities					Relative standard uncertainty (%)	
	Mo / Mo23	Mo / Mo25	Mo / Mo28	Mo / Mo30	Mo / Mo35	Type A	Type B
Air attenuation *k*_a_^a^	1.055	1.051	1.047	1.043	1.040		0.01
Scattered radiation *k*_sc_	0.9949	0.9950	0.9950	0.9951	0.9952		0.07
Electron loss *k*_e_	1.000	1.000	1.000	1.000	1.000		0.05
Ion recombination *k*_s_	1.000	1.000	1.000	1.000	1.000	0.04	
Humidity *k*_h_	0.998	0.998	0.998	0.998	0.998		0.03

a These are nominal values for *T* = 293.15 K and *p* = 101 325 Pa. Each measurement is corrected using the air temperature and pressure measured at the time.

**Table 5b:** Correction factors used in the comparison for the NIST Attix mammography standard

Correction factor	Reference radiation qualities				Relative standard uncertainty (%)	
	Mo / Mo25x	Mo / Mo28x	Mo / Mo30x	Mo / Mo35x	Type A	Type B
Air attenuation *k*_a_^a^	1.021	1.024	1.021	1.020		0.01
Scattered radiation *k*_sc_	0.9955	0.9956	0.9956	0.9957		0.07
Electron loss *k*_e_	1.000	1.000	1.000	1.000		0.05
Ion recombination *k*_s_	1.000	1.000	1.000	1.000	0.04	
Humidity *k*_h_	0.998	0.998	0.998	0.998		0.03

a These are nominal values for *T* = 293.15 K and *p* = 101 325 Pa. Each measurement is corrected using the air temperature and pressure measured at the time.

**Table 6a:** Correction factors used in the comparison for the PTB PK100 standard: BIPM reference radiation qualities (PTB F-series)

Correction factor	Generating potential (kV)					Relative standard uncertainty (%)	
	10	25	30	50a	50b	Type A	Type B
Air attenuation *k*_a_^a^	1.2274	1.0340	1.0485	1.0046	1.0095	0.05	0.05
Scattered radiation *k*_sc_^b^	0.9854	0.9901	0.9893	0.9939	0.9927		0.05
Electron loss *k*_e_	1.0000	1.0000	1.0000	1.0000	1.0000		0.05
Volume recombination *k*_sv_	1.0014	1.0017	1.0026	1.0012	1.0018	0.05	0.05
Initial recombination *k*_si_	1.0005	1.0005	1.0005	1.0005	1.0005		0.05
Guard srip attenuation *k*_ap_	1.0393	1.0075	1.0110	1.0015	1.0027	0.05	0.05
Aperture edge transmission *k*_1_	0.9998	0.9991	0.9992	0.9971	0.9979		0.05
Field distortion *k*_d_	0.9920	0.9920	0.9920	0.9920	0.9920		0.15
Wall transmission *k*_p_	1.0000	1.0000	1.0000	1.0000	1.0000	0.05	
Humidity *k*_h_	0.9980	0.9980	0.9980	0.9980	0.9980		0.05

a These are nominal values for *T* = 293.15 K and *p* = 101 325 Pa. Each measurement is corrected using the air temperature and pressure measured at the time.

b This correction includes the re-absorption of scattered radiation and of fluorescent photons.

**Table 6b:** Correction factors used in the comparison for the PTB PK100 standard: Mammography radiation qualities, non-attenuated (PTB MMV-series)

Correction factor	Generating potential (kV)					Relative standard uncertainty (%)	
	20	25	28	30	35	Type A	Type B
Air attenuation *k*_a_^a^	1.0333	1.0275	1.0256	1.0250	1.0232	0.05	0.05
Scattered radiation *k*_sc_^b^	0.9899	0.9904	0.9906	0.9907	0.9909		0.05
Electron loss *k*_e_	1.0000	1.0000	1.0000	1.0000	1.0000		0.05
Volume recombination *k*_sv_	1.0001	1.0004	1.0004	1.0005	1.0005	0.05	0.05
Initial recombination *k*_si_	1.0005	1.0005	1.0005	1.0005	1.0005		0.05
Guard srip attenuation *k*_ap_	1.0068	1.0056	1.0052	1.0051	1.0047	0.05	0.05
Aperture edge transmission *k*_1_	0.9997	0.9997	0.9996	0.9996	0.9996		0.05
Field distortion *k*_d_	0.9920	0.9920	0.9920	0.9920	0.9920		0.15
Wall transmission *k*_p_	1.0000	1.0000	1.0000	1.0000	1.0000	0.05	
Humidity *k*_h_	0.9980	0.9980	0.9980	0.9980	0.9980		0.05

a These are nominal values for *T* = 293.15 K and *p* = 101 325 Pa. Each measurement is corrected using the air temperature and pressure measured at the time.

b This correction includes the re-absorption of scattered radiation and of fluorescent photons.

**Table 6c:** Correction factors used in the comparison for the PTB PK100 standard: Mammography radiation qualities, attenuated (PTB MMH-series)

Correction factor	Generating potential (kV)					Relative standard uncertainty (%)	
	20	25	28	30	35	Type A	Type B
Air attenuation *k*_a_^a^	1.0138	1.0116	1.0110	1.0106	1.0098	0.05	0.05
Scattered radiation *k*_sc_^b^	0.9916	0.9919	0.9920	0.9921	0.9923		0.05
Electron loss *k*_e_	1.0000	1.0000	1.0000	1.0000	1.0000		0.05
Volume recombination *k*_sv_	1.0001	1.0004	1.0004	1.0005	1.0005	0.05	0.05
Initial recombination *k*_si_	1.0005	1.0005	1.0005	1.0005	1.0005		0.05
Guard srip attenuation *k*_ap_	1.0029	1.0024	1.0023	1.0023	1.0021	0.05	0.05
Aperture edge transmission *k*_1_	0.9996	0.9995	0.9995	0.9994	0.9994		0.05
Field distortion *k*_d_	0.9920	0.9920	0.9920	0.9920	0.9920		0.15
Wall transmission *k*_p_	1.0000	1.0000	1.0000	1.0000	1.0000	0.05	
Humidity *k*_h_	0.9980	0.9980	0.9980	0.9980	0.9980		0.05

a These are nominal values for *T* = 293.15 K and *p* = 101 325 Pa. Each measurement is corrected using the air temperature and pressure measured at the time.

b This correction includes the re-absorption of scattered radiation and of fluorescent photons.

**Table 7:** Relative standard uncertainties (in %) associated with the standards

Source of uncertainty	NIST Attix		NIST Ritz		PTB	
	Type A	Type B	Type A	Type B	Type A	Type B
Ionization current	0.13	0.06	0.05	0.06	0.10	0.06
Volume	0.01	0.07	0.04	0.01		0.06
Positioning		0.01		0.01		0.01
Correction factors (excl. *k*_h_)	0.04	0.09	0.03	0.14	0.10	0.20
Physical constants		0.15		0.15		0.15

${\dot{K}}_{\text{LAB}}$	0.14	0.20	0.07	0.22	0.14	0.26

	0.24		0.23		0.30	

**Table 8:** Relative standard uncertainties (in %) associated with the calibration of the transfer ionization chambers

Source of uncertainty	NIST Attix		NIST Ritz		PTB	
	Type A	Type B	Type A	Type B	Type A	Type B
Air-kerma rate ${\dot{K}}_{\text{LAB}}$	0.14	0.20	0.07	0.22	0.14	0.26
Ionization current	0.09	0.06	0.05	0.06	0.10	0.06
Positioning		0.01	0.01			0.04
Monitor normalization						0.05
Air density correction						0.04

*N_K_*_,LAB_	0.17	0.21	0.09	0.23	0.17	0.28

	0.27		0.24		0.33	

**Table 9:** Relative standard uncertainties (in %) associated with the comparison results *R_K_*

Source of uncertainty	Type A	Type B
Comparison result *R_K_*	0.24	0.28
	0.37	

**Table 10a:** Measured PTB results for the calibration of the NIST transfer chambers at both laboratories

Reference radiation	NIST transfer chambers calibration coefficients (10^7^ Gy C^−1^)			
(*polarity of charge*)	A11 SN114 (*negative*)	A11 SN114 (*positive*)	A11 SN114 (*negative*)	A11 SN114 (*positive*)
BIPM10	3.326	3.315	1.341	1.347
BIPM25	3.268	3.245	1.259	1.265
BIPM30	3.262	3.243	1.269	1.277
BIPM50a	3.203	3.162	1.207	1.213
BIPM50b	3.247	3.210	1.221	1.226
MMV 20	3.259	3.239	1.254	1.263
MMV 25	3.267	3.245	1.245	1.254
MMV 28	3.271	3.250	1.243	1.251
MMV 30	3.273	3.252	1.242	1.251
MMV 35	3.276	3.254	1.239	1.248
MMH 20	3.293	3.250	1.235	1.240
MMH 25	3.281	3.250	1.225	1.233
MMH 28	3.278	3.249	1.222	1.230
MMH 30	3.274	3.245	1.221	1.228
MMH 35	3.268	3.240	1.218	1.226

**Table 10b:** Measured PTB results for the calibration of the NIST transfer chambers at both laboratories

Reference radiation	NIST transfer chambers calibration coefficients (10^7^ Gy C^−1^)			
(*polarity of charge*)	A11 SN114 (*negative*)	A11 SN114 (*positive*)	A11 SN114 (*negative*)	A11 SN114 (*positive*)
BIPM25	3.260	3.245	1.258	1.262
BIPM30	3.247	3.231	1.260	1.269
BIPM40	3.202	3.186	1.209	1.218
BIPM50a	3.207	3.183	1.209	1.216
BIPM50b	3.262	3.237	1.225	1.232
Mo/Mo23	3.264	3.252	1.247	1.262
Mo/Mo25	3.267	3.246	1.244	1.254
Mo/Mo28	3.260	3.248	1.250	1.252
Mo/Mo30	3.271	3.246	1.240	1.245
Mo/Mo35	3.270	3.242	1.243	1.242
Mo/Mo25x	3.291	3.269	1.234	1.240
Mo/Mo28x	3.293	3.268	1.227	1.236
Mo/Mo30x	3.291	3.261	1.227	1.229
Mo/Mo35x	3.262	3.249	1.219	1.230

**Table 11:** Results of the comparison for each of the transfer chambers shown as the ratio of the average calibration coefficient *N*_K, NIST_ / *N*_K, PTB_ for both chambers, as well as the mean

Reference radiation	A11 SN114	A15 SN103	Mean *N_K_*_, NIST_ / *N_K_*_, PTB_
BIPM25	0.999	0.998	0.998
BIPM30	0.996	0.993	0.995
BIPM50a	1.004	1.002	1.003
BIPM50b	1.007	1.004	1.005
Mo/Mo25	1.000	0.999	1.000
Mo/Mo28	0.998	1.003	1.001
Mo/Mo30	0.999	0.997	0.998
Mo/Mo35	0.997	0.999	0.998
Mo/Mo25x	1.004	1.006	1.005
Mo/Mo28x	1.005	1.005	1.005
Mo/Mo30x	1.005	1.003	1.004
Mo/Mo35x	1.000	1.002	1.001

## Footnotes

1 Certain commercial equipment, instruments, or materials are identified in this paper to foster understanding. Such identification does not imply recommendation or endorsement by the National Institute of Standards and Technology, nor does it imply that the materials or equipment identified are necessarily the best available for the purpose
